# Correction: 27 T ultra-high static magnetic field changes orientation and morphology of mitotic spindles in human cells

**DOI:** 10.7554/eLife.28212

**Published:** 2017-05-04

**Authors:** Lei Zhang, Yubin Hou, Zhiyuan Li, Xinmiao Ji, Ze Wang, Huizhen Wang, Xiaofei Tian, Fazhi Yu, Zhenye Yang, Li Pi, Timothy J Mitchison, Qingyou Lu, Xin Zhang

Zhang L, Hou Y, Li Z, Ji X, Wang Z, Wang H, Tian X, Yu F, Yang Z, Pi L, Mitchison TJ, Lu Q, Zhang X. 2017. 27 T ultra-high static magnetic field changes orientation and morphology of mitotic spindles in human cells. *eLife* **6**:e22911. doi: 10.7554/eLife.22911.

Published 28, February 2017

In the published article we missed an important reference (Valles et al. 2002) so that we need to make the following changes in the Abstract, Introduction and Discussion.

The reference is: Valles JM, Wasserman S, Schweidenback C, Edwardson J, Denegre JM, Mowry KL. 2002. Processes That Occur before Second Cleavage Determine Third Cleavage Orientation in *Xenopus*. Experimental Cell Research **274**:112–118. doi:10.1006/excr.2001.5456.

In the second sentence of the abstract, the text “However, whether mitotic spindle in cells can be aligned by magnetic field has not been experimentally proved.” should be replaced with “However, whether mitotic spindle in mammalian cells can be aligned by magnetic field has not been experimentally proved.”

Also in the abstract, “the biological effects of SMF of above 20 T (Tesla) have never been reported.” should be changed to “the biological effects of SMF of above 20 T (Tesla) on mammalian cells have never been reported.” In addition, “This is the first reported study that investigated the cellular responses to ultra-high magnetic field of above 20 T.” should be changed to “This is the first reported study that investigated the mammalian cellular responses to ultra-high magnetic field of above 20 T.” The same erroneous statement appeared one more time in the first sentence of the second paragraph of the Discussion and should be corrected there as well.

In the third paragraph of the Introduction, “which was theoretically proved later by Valles (Valles, 2002), but no experimental evidence has been provided so far to prove this hypothesis.” should be changed to “which was theoretically and experimentally proved later (Valles, 2002; Valles et al., 2002).” The last sentence “Therefore, the theoretical prediction of the SMF effect on spindle orientation in cells has not been experimentally tested.” should be changed to “Therefore, although the theoretical prediction of the SMF effect on spindle orientation in cells has been experimentally tested in Xenopus embryo, how the spindles in mammalian cells, which have very different structure than Xenopus embryo, are affected is still unknown.”

In the third paragraph of the Discussion, “Based on theoretical calculation, Valles also proposed that … align within it (Valles, 2002).” should be changed to “Based on theoretical calculation and experimental validation, Valles and his colleagues proposed that … align within it (Valles, 2002; Valles et al., 2002).”

These changes do not affect the key points and conclusions of the original paper.

